# Living with Schizophrenia: the role of interpersonal relationships

**DOI:** 10.1007/s44202-023-00071-9

**Published:** 2023-06-13

**Authors:** Isaac Tetteh Commey, Jerry Paul K. Ninnoni, Evelyn Asamoah Ampofo, Daniel Miezah

**Affiliations:** 1 Department of Mental Health, School of Nursing and Midwifery, College of Health and Allied Sciences, University of Cape Coast, PMB, Cape Coast, Ghana; 2 Department of Maternal and Child Health, School of Nursing and Midwifery, College of Health and Allied Sciences, University of Cape Coast, PMB, Cape Coast, Ghana; 3 Department of Education and Psychology, Faculty of Educational Foundations, College of Education Studies, University of Cape Coast, PMB, Cape Coast, Ghana

**Keywords:** Ghana, Interpersonal relationship, Schizophrenia, Stigma

## Abstract

People with a chronic condition such as schizophrenia encounter significant challenges interacting with their immediate environment. However, there is little data exploring interpersonal relationships between people living with schizophrenia and their families and healthcare providers, particularly in developing countries. This study investigated the interpersonal relationship experiences of persons with schizophrenia in Southern Ghana. The study adopted a descriptive phenomenological approach using the purposive sampling technique to recruit nine (9) persons living with schizophrenia. Data were collected using semi-structured in-depth, face-to-face interviews and analysed using a descriptive phenomenological data analysis framework. Five themes emerged; three described positive interpersonal relationships, and two negative interpersonal relationships existed in participants with schizophrenia. The study revealed a poor interpersonal relationship between study participants and the public. Stigma was implicated as a factor responsible for the negative interpersonal relationships between study participants and people outside their immediate families.

## Introduction

Schizophrenia typically manifests in early adulthood and becomes chronic over time [1]. Schizophrenia has significant implications for both health service planning and risk-factor epidemiology. Persons with schizophrenia experience significant difficulties in their social functioning, including problems getting jobs, marriage and living independently. This leads to reduced quality of life [2]. This study explored the interpersonal relationship of persons living with schizophrenia.

It is claimed that an essential feature of schizophrenia is impaired social functioning [3]. The worsening of social relations remains a trademark of schizophrenia, with social isolation and withdrawal forming part of its clinical manifestations [4]. Stigmas, such as social rejection and problematic family relationships, are some negative consequences of living with schizophrenia [5]. Many family members of individuals with schizophrenia are confronted with the symptoms of the illness and the social disruptions associated with it daily. The stigma and shame associated with mental ill-health can significantly reduce social networks, even for carers [6]. The importance of social support in reducing distress and encouraging more proactive coping among carers has been established [7].

There have been several interventions for people with schizophrenia to maintain a good quality of life in their communities [8]. However, despite these interventions, a more significant proportion of them often encounter reoccurrence of clinical manifestations and struggle to maintain a good quality of life [9]. In particular, in low and middle-income countries with poorer health services and social support, people with schizophrenia struggle to integrate with society. It is argued that a harmonious and therapeutic relationship is a prerequisite for maintaining resilience among people with chronic conditions such as schizophrenia [10]. An interpersonal relationship may be regarded as an intense, close connection between two or more people ranging from short to long-term [11]. The situation may contrast with family or kinship relations, friendship, marriage, relations with acquaintances, work, and places of fellowship or worship [12]. Relationships among people may be determined by law, custom, or joint agreement, forming the foundation of social groups and society or organisation. This association may be based on love, unity, mutual support, consistent interactions, social connection and commitment [12]. Research suggests that good interpersonal relationships are suitable for healthy mental health and well-being [13–15]. Sociological and psychological literature has contended that social ties can cushion stress or anxiety associated with schizophrenia [16].

A significant body of literature from the ecological perspectives on health promotion has revealed that social support is associated with increased psychological well-being and a lower probability of physical illness [17]. The benefits provided by effective interpersonal relationships play an essential role in determining people's adaptive functioning and health outcomes [18]. For example, a positive interpersonal relationship promotes self-esteem, giving hope and a sense of well-being among persons with schizophrenia [19]. On the other hand, a negative relationship decreases the individuals’ self-esteem, makes them withdrawn, and affects their ability to contribute meaningfully to society [20]. A recent study in Ghana, suggested that people with schizophrenia are stigmatised in their communities [21] and this will influence their interpersonal relationships. Still, no known study examined people with schizophrenia experiences regarding interpersonal relationships.

In Ghana, schizophrenia has been investigated from the viewpoints of healthcare practitioners, carers and families of persons with schizophrenia [22–27]. However, studies exploring the interpersonal relationship of persons with schizophrenia in Ghana are lacking. It is argued that the service user is the primary source of any information regarding their lived experiences and the best person to define recovery and relapse [28–30]. Therefore, there is a need to explore the interpersonal relationship of individuals living with schizophrenia to discover their experiences to inform policies regarding their support needs. This study aimed to bridge this research gap by exploring the experiences of people living with schizophrenia regarding interpersonal relationships in Ghana.

## Methods

### Study design

A descriptive phenomenological design was employed in this study due to the sensitive nature of the subject matter and the need to elicit participants’ lived experiences where ideas are generated from a rich data using an inductive approach [31]. Descriptive phenomenology is mainly employed in qualitative research when little is known about a phenomenon [32], especially in Ghana, where little is known about this phenomena.

### Study setting

The study explored the interpersonal relationship of persons living with Schizophrenia in Cape Coast (Southern Ghana). This was a community-based study; participants were recruited from the Cape Coast metropolis. The study was designed to recruit only persons who were living with schizophrenia in their homes and supported by community psychiatric nurses. Five community psychiatric units are attached to five healthcare facilities in the Cape Coast Metropolis. These facilities offer community mental healthcare to discharge persons with mental illness in the metropolis. After receiving ethical clearance for the study, the researchers contacted the nurse in charge of the outpatient unit of each of the five community psychiatry units to explain the purpose of the study to them. The contact nurse in each community psychiatric unit identified persons who met the inclusion criteria for the study by assessing them using a Mental Status Examination guide, informed them about the study and sought their permission to give their phone numbers to me. After receiving the contact numbers of potential participants, all those who met the inclusion criteria were put together into a single document (journal) to form the population under study.

The lead investigator personally contacted them one by one through telephone calls and home visits to explain in detail what the study was about and invited them to participate. The interview time, date and place were negotiated with persons who consented to be part of the study. All participants who were contacted chose to be interviewed in their homes. The rationale for recruiting from the local community of the target participants was to gain individual views and generate findings which were likely to be relevant to local mental health care provision.

### Study population

The target population for the research included all persons living within the Cape Coast Metropolis with schizophrenia and receiving mental health services. These persons were once diagnosed and managed by psychiatric mental health experts and discharged into various communities within the metropolis. Only participants who could provide informed consent were included. A purposive sampling technique was used to recruit individuals with schizophrenia who can share rich experiences regarding interpersonal relationships.

### Data collection instrument and procedure

Data was collected using a semi-structured interview schedule developed based on human interpersonal relationships. The researchers conducted nine face-to-face tape-recorded interviews with persons living with schizophrenia until saturation, when it was observed that no significant new information was being gathered from study participants regarding the phenomena [33]. Although, the interviews were not triangulated, the three other authors listened to the audio tape recordings from the beginning of the data collection to ensure that the data collected captured the study participants' views that focused primarily on the phenomenon under study and thus increased the trustworthiness of the study [34]. The interview guide sought to inquire into the interpersonal relationship experience of study participants. Leading questions such as “how long have you lived with schizophrenia?”, “What is your relationship with your immediate environment?” and how do people around you relate with you?” were asked. Some probing questions asked included “, can you share your relationship with people you interact daily with?” your religious group members? Your parents? Spouse? Children? Mental health professionals?

An average duration of 45 min was spent with each participant during the interview sessions. The data was secured on a password-protected computer and only accessible by the lead researcher.

### Trustworthiness

This was ensured by adopting approaches including credibility, transferability, dependability and confirmability [34]. Credibility, the accurate and truthful depiction of a participant's lived experience, was achieved in this study through continued engagement and observations to describe the phenomenon’s context and minimise distortions that might interfere with the data. Transferability was also enhanced by using the purposive sampling method to purposefully select individuals who met the inclusion criteria and were willing to provide a detailed description to come out with robust data with a wide range of information through clear and accurate descriptions of participants’ lived experiences of schizophrenia by continuously returning to the texts. Dependability was also achieved by having an expert qualitative nursing researcher review the transcribed material to validate the themes and descriptors identified. Co-authors did this with a wealth of experiences in qualitative text. The aim here was that both analysts agree on the themes and meanings within the transcribed material. Finally, confirmability was achieved by documenting the procedures for checking and rechecking the data throughout the study. Consequently, the collected and analysed data were presented to the study participants to ascertain whether the narrative was accurate and a true reflection of their experiences.

### Ethical considerations

The study was granted ethical clearance by the Institutional Review Board of the University of Cape Coast (UCCIRB/CHAS/2020/37) after demonstrating how conditions of informed consent, anonymity, privacy and confidentiality will be maintained in the study. Therefore, the study was carried out based on the guidelines stipulated by the ethical review board of the University of Cape Coast.

### Data analysis

All interviews were transcribed verbatim into written text for analysis. Colaizzi's seven steps in descriptive phenomenological data analysis [35] were followed.

Firstly, familiarisation with the data took place. To achieve this, the researcher familiarised himself by submerging in the data and carefully reading each transcript several times to gain a complete understanding. Second, significant statements directly relevant to the phenomenon under investigation were identified. An example of a powerful statement regarding the impact of schizophrenia included, *“……. this illness has affected my work because if it had not been this illness, I know where I wanted to be in future would not have been this (Favour, 32 years, Female Fashion designer; 28th May 2020).*

The third step focuses on formulating meanings by identifying meanings relevant to the phenomenon. Several meanings were developed from the significant statements generated in the second step. Examples of formulated meanings included; the economic impact of schizophrenia, labelling by people, pastoral support, family support available, reoccurrence of the condition etc.

The fourth step focuses on the clustering of themes. The researcher clustered the formulated meanings into common themes across all participants’ accounts, which were significant to the phenomenon under study. The pieces that sought to describe the interpersonal relationship experience of people were isolated at this stage. At this stage, bracketing was ensured so that the researcher’s assumptions, presuppositions and what is already known about the phenomenon were put aside during the data analysis [36, 37]. All that was known about schizophrenia and individuals who have lived with the condition over time was written down. The researcher made sure he did not jump to conclusions based on what was known about the phenomenon before the study. As much as possible, the study participants’ voices were given the needed attention during the study. The fifth step is about developing a detailed description of the themes. The researcher wrote a full and inclusive definition of the phenomenon incorporating all the pieces produced in step four. The sixth step of the analysis talks about the production of fundamental structure. The researcher condensed the detailed description down to a short, dense statement that captured just those aspects deemed essential to the design of the phenomenon. Essentially, the sixth step is where the study report was generated. Finally, the seventh step is to seek verification of the fundamental structure. This is where the fundamental structure statement (essay) was returned to all study participants to ask whether it captured their experience. Earlier steps in the analysis were modified in light of this feedback.

## Results

The table below shows that seven out of nine participants were single, while two were married. On the age range of participants, the data revealed that one of the participants was in the age range of 20 -30 years. This was followed by three in the age range of 31–40 years. Also, the remaining five participants were in the age range of 41 years and above. Concerning the gender of the participants, six were females, whilst three were males.

Eight participants were Christians, except one who indicated that he was a Muslim. Two had formal education up to the junior high school level. Again, four respondents were educated up to the senior high school level, whilst the remaining three participants verbalised that they had tertiary education. Finally, two respondents indicated that they had lived with the condition for 21 years and above while five respondents had lived with schizophrenia for 11 to 20 years. The remaining two were diagnosed with schizophrenia 10 years ago (see Tables 1, 2).

**Table 1:** Results of demographic data (N = 9)

Demographic information	Frequency	Percentage (%)
Marital status		
Married	2	22.22
Single	7	77.78
Age (in years)		
20–30	1	11.11
31–40	3	33.33
41and above	5	55.56
Gender		
Male	3	33.33
Female	6	66.67
Religion		
Christianity	8	88.89
Islamic	1	11.11
Educational background		
Junior High	2	44.44
Senior High	4	44.44
Tertiary	3	11.11
No. of years with Schizophrenia		
1–10	2	22.22
11–20	5	55.56
21 and above	2	22.22

Source: Field work

**Table 2:** Overview of Emerged Themes on Interpersonal Relationships

Positive	Negative
Relationship with family	Spousal relationship
Relationship with support organisations and society	Relationship with working colleagues
Relationship with mental healthcare providers	

Source: Field work

Five key themes emerged from the interpersonal relationship experiences of study participants. These were classified under positive and negative relationships below;

### Positive interpersonal relationships

Positive interpersonal relationships are harmonious and therapeutic interactions among people within a geographical area. This type of relationship is likely to improve the growth of chronic conditions and give them a sense of belonging. Study participants recounted the following positive interpersonal relationships. These include:

Relationship with family; Relationship with support organisations and society; Relationship with Mental and Healthcare Providers.

### Negative interpersonal relationships

Negative interpersonal relationships comprise the maladaptive patterns of association between two or more people within a specified area. This is likely to affect the daily functioning of the individuals and, thus, lowers their self-esteem. In this study, the following were some of the negative relationships that participants identified:

- Spousal relationship.
- Relationship with Working colleagues.

###  Relationship with the family

This sub-theme explores the relationship between study participants and their immediate family members. The study finding indicated that participants could maintain good and positive relationships with people around them. The study participants and family members have good relations with them and have been supportive since they were diagnosed with schizophrenia. Mothers had a good and positive relationship with participants compared to other family members. The accounts by participants revealed that these family members, especially mothers, always show love towards them and understand their condition. Below are narratives of how participants recounted the relationship they shared with family members:

“Family members, especially my parents and siblings, did not abandon me when I was diagnosed with this condition. They rather came to my aid to reassure me of their support…they inspired and supported me with some basic needs for my upkeep. My mother is my best friend. She always calls me to reaffirm that she is there for me whenever I need her.……….” (Participant 1, 58 years, Male Teacher; 25th May 2020).

“…. My mother, for example, understands me better than any other relationship. She encourages me to stick to the treatment plan… anytime I feel sad about this condition, my siblings and mother come to my aid and tell me they are there for me. Our relationship keeps me healthy all this while” (Participant 2, 35 years, Female Caterer; 26th May 2020).

It is worth noting that, although some (six) participants verbalised having a positive relationship with family members right from the first day of their diagnosis, three (3) of the participants indicated having challenges initially with family members when they first got to know that they were living with schizophrenia. A typical example is the narrative below:

“ …. At first, my family members, including my parents, thought someone had cursed me; that is why I have become mentally ill. As a result, they did not want to eat with me when I came home. They believed that if they ate with me, they could also develop a mental health condition. So, it marred our peaceful relationship. It took the intervention of nurses who came to my house to educate them on my condition before they agreed to relate well with me.” (Participant 4, 44 years, Female businesswoman; 22nd May 2020).

### Spousal (marital) relationship

On the issue of marriage, the study sought to find out how living with schizophrenia has influenced participants’ experiences with marriage and marital relationships. Findings from the survey revealed that two out of the nine participants were married and were happily living with their spouses. In addition, the married ones indicated that they had a positive marital relationship.

“…. You see, my current wife sometimes makes funny comments about me. She is very jovial and has a great sense of humour. That is how she has been ever since we married. However, she does not use my condition to insult or look down on me. On the contrary, we share jokes …you can see that we are living peacefully here.” (Participant 1, 58 years, Male Teacher; 25th May 2020).

*Participant 5*, (38 years; Female aviation security personnel; 9th June 2020) added;

“….. You will enjoy a peaceful marital life if you get someone who understands you. In my case, my husband understands me. He has been my source of encouragement despite living with this condition. We have a cordial relationship because he knows my situation well”. He interacts with me regularly to know how I am faring daily….”

Two of the seven participants who were unmarried at the time of data collection explained how potential suitors withdrew from them upon discovering their mental illness.

“…………. then he said, is this a mental drug? I responded yes. Then he asked me if I had a mental illness, to which I said yes. Since then, he has neither visited me nor called me again. That was the end of our relationship, my brother” (Participant 6, 41 years, Female dressmaker; 18th June 2020).

“……This condition scares men from me… they don’t want to marry someone with mental illness. They do not want to associate with people with this illness because they think we are mad… I can’t even mingle with guys around my area. They are interested in me, but they are afraid to come close because of this condition” ( Participant 2, 42 years, Sales personnel, 21st June 2020)

### Relationship with support organisations and society

This sub-theme focused on the interpersonal relationship between participants and supported organisations, including societies available to persons with schizophrenia.

To participants, their primary source of support was family. However, some cordial relationship exists between them and some organisations and societies. According to the respondents, these organisations or institutions included the church and social welfare department. They periodically go to their aid to check up on them, provide them with emotional and psychological support, and provide basic needs to supplement what they receive from family and mental healthcare practitioners. The following responses illustrate this assertion.

“…the social welfare department came to find out how I was faring in my house and offered me some financial support last year. They came to visit me with some mental health nurses, and then I told them of my challenges in life.” They

gave me their contact to call them anytime I want someone to share my problems with….” (Participant 7, 32 years, Female Fashion designer; 28th May 2020).

“… Anytime I go to my fellow Muslim brothers, they offer me the needed help in terms of money, food items and prayers. They always want to see me around them. I experience brotherly love anytime I go to the mosque to pray.”.” (Participant 9, 41 years, Male Fisherman; 25th May 2020).

### Relationship with working colleagues

In exploring the interpersonal relationship between clients with schizophrenia and their working colleagues, study participants noted that living with schizophrenia has affected their relationship with their colleagues at work and their work output. Participants verbalised that their colleagues did not understand the course of their condition and its treatment outcome. They further indicated that this incivility at work sometimes makes them lose their temper and misbehave. Some subsequently lost their job due to the hostile working environment in which they found themselves.

“As a teacher, I always had some exchanges with colleague staff. They don’t understand my situation, so they sometimes ridicule me. Sometimes, I had to question them why they didn’t want to include me in activities. Their behaviour tells me they don’t want to be in the same group. Anytime I question authorities, they tell me I am too troublesome…when the principal notices that I am frustrated, she asks me to go home and rest.” (Participant 3, 43 years, Female Teacher; 22nd June 2020).

*Participant 5*, (38 years; Female aviation security personnel; 9^th^ June 2020) added;

“…I used to work in the aviation department as a security staff. The staff appeared ignorant of my condition; sometimes, their behaviour towards me was bad. They discriminate towards me…... At times they will go to meetings without informing me. I don’t know if they think I will harm them or not. I lost my job subsequently because of this condition. It was a painful experience. I have not been able to secure any job till now.”

### Relationship with mental healthcare providers

Participants also described their relationship experiences with mental health practitioners like nurses, doctors and other health workers in mental health facilities. According to the participants, caregivers like nurses and doctors show much concern about their conditions, especially when visiting mental health facilities. In addition, participants revealed that nurses and doctors often complement what family members do for them.

*Participant 5* (38 years; Female aviation security personnel; 9^th^ June 2020) had this to say about her relationship with caregivers:

“.. As for the nurses and doctors always pray and wish I become completely free from this illness. This is because they call me on the phone to check up on me every week. …. The nurses are excellent and caring. They are even part of the reason why I am feeling fine. They have been supportive, and I am proud of them.”

*Participant 8* (20 years, Male student; 20th June 2020) also added that:

“……. Look at the road you used. It isn't very good, but these nurses always want to come and interact with me about my welfare. The nurses do not joke with me, especially when I visit the mental health facility for a check-up. I don't know where I would have been by now had it not been for the support of the nurses at the mental facilities.…. I visit the clinic outside of my review days to interact with them about my condition ……. They have been a blessing to me all these years.”

## Discussion

This study aimed to explore the interpersonal relationship experiences among nine service users living with schizophrenia in Cape Coast, Ghana. With this aim, the researchers asked study participants to share their rich and natural interpersonal relationship experiences whilst living with schizophrenia. This study delved into the private lives of individuals with schizophrenia and explored vital issues centred on how they interact with people within their immediate environment.

Regarding the experiences of building and maintaining a relationship with family members and caregivers, the findings showed that cordial relations exist between the study participants and their family members, including caregivers. This study shows that individuals with the condition who participated in the survey have a friendly association with their family members and receive essential support. Since their relatives are compassionate, this assistance helps to reduce the incidence of stigma on individuals with schizophrenia. This harmonious relationship also boosts their self-esteem, reducing psychological problems that may lead to relapse. In a related development, Davis [38] explored the experiences of individuals with schizophrenia in Denmark. He found that people with schizophrenia had united and close interactive living with immediate family members. Furthermore, it was found in Turkey that persons with schizophrenia had an improved relationship with their families and experienced acceptance and love from their relatives [39]. Therefore, it can be concluded that the family is a source of emotional support to persons with schizophrenia because of this supportive family-client relationship.

Regarding marital relationships, participants’ accounts suggest that the diagnosis of mental illness can negatively affect a person’s opportunity to attract potential suitors and have a possible marital relationship. Many people have developed some fear towards mental illness, particularly schizophrenia. It has been investigated and found that schizophrenia is a neuropsychiatric disorder associated with divorce [39]. In China, marriage outcomes among couples with schizophrenia have been poor [39]. They indicated that acrimonious and hostile spousal relationships existed among persons with a history of schizophrenia and married to their partners without any traces of the condition. Remissions and relapses, coupled with uncertainty about treatment outcomes of schizophrenia, put some fear in individuals who fall in love with people with a history of schizophrenia. This has made it difficult for people to unite with someone with a history of this condition. Some have suffered a divorce after discovering that their partners live with schizophrenia. It can be concluded that being diagnosed with schizophrenia can affect one’s chances of having a successful marital relationship.

Moreover, the study findings indicated that schizophrenia affects the working life of study participants. An uncivil and unkind relationship existed between clients and their operational colleagues. Sometimes, colleagues at work exclude these individuals from meetings, staff activities, planning, and groupings [40]. Many organisations have been prevented from naming ceremonies, funerals and other activities involving their working colleagues because they live with schizophrenia [40]. Individuals with schizophrenia have had to isolate themselves from the public due to this social exclusion and stigmatisation in the public domain. This sometimes affects the work output of persons with schizophrenia. Again, it affects the livelihood of the individual with schizophrenia because employment provides income and improves social contact for people with schizophrenia, fosters financial benefits and reduces the burden on the family. This, therefore, presupposes that participants with schizophrenia may not be able to enjoy these benefits since they do not have the peace of mind to bring them to their workplaces.

Also, a healthy relationship existed between participants and mental health nurses, which may have contributed to the maintenance of resilience among study participants. It can be argued that, due to these professionals' knowledge of the condition, their engagement with participants has been cordial, as verbalised by clients (participants). Persons with schizophrenia build friendly relationships with mental health professionals like doctors and nurses [41]. This enables them to receive more significant support, especially when they visit health facilities. Even though the findings of this study find support in the literature as discussed so far, other previous studies [40, 41, 42] contradict the current findings. For example, [43] concluded that health professionals ignored patients diagnosed with schizophrenia in Tasmania. Evidence points to the fact that patients with schizophrenia in Britain faced many challenges, including hostile interactions with frontline health professionals, especially nurses [40]. The contradiction here may be possibly due to the study setting. Also, health professionals in Ghana are likely to benefit from proper orientation about caring for mental disorders at the various mental health facilities.

In Connection to relationships with institutions or societies, it was evident that the church and the social welfare department within the metropolis had some connection with participants. It suggests that these institutions provide welfare services for individuals with chronic conditions, of which schizophrenia is no exception. Participants had the chance to walk to their offices for any form of assistance when necessary. This support augments what the family and healthcare providers give to these clients in their quest to help them maintain resilience.

Similarly, in China, Wang et al. [42] concluded that government and religious bodies provide adequate and regular support for people with schizophrenia. These organisations offer them monies and other basic needs to complement the support received from their family members. Institutions, including social welfare, play a vital role in persons with chronic conditions such as schizophrenia. Therefore, it is not out of place that studies have identified their cordial relationship with persons with schizophrenia.

## Conclusions

This study has shed light on how participants living with schizophrenia interact with people in their lives. A positive relationship existed between participants and their family members, institutions and mental health professionals. However, participants had dysfunctional marital relationships and poor working relationships with colleagues. In addition, participants verbalised being discriminated against at work by colleagues. These findings are not vividly different from those reported in previous literature; however, the interpersonal needs of people with schizophrenia may differ from a cultural point of view.

## Study limitations

Qualitative research is often criticised for needing more generalizability and requiring more reliance on researchers' subjective interpretations. Therefore, the results of this study cannot be generalised as the true reflection of all persons living with schizophrenia in Ghana. Again, considering the study’s sample size (9 persons), the study participants' opinions will not be generalised to represent the views of all persons living with schizophrenic illness in the Cape Coast Metropolis. It was, however, not the researchers' aim to make generalisations but to understand and describe the interpersonal relationship experiences of persons living with schizophrenia in the Cape Coast Metropolis.

## Recommendations

Based on the findings of the study, the following recommendations were made:

### Nursing practice

Community mental health nurses need to intensify their home visits to individuals with schizophrenia in their catchment area to support clients who encounter challenges with poor interpersonal relationships and to build confidence and social skills.

### Future directions

Research can be conducted on gender differences in the experiences of interpersonal relationships among persons with schizophrenia to determine if differences exist between males and females living with schizophrenia. In addition, similar nationwide research by any interested organisation or the Ghana Health Service will be commendable. Such research findings will either confirm this study's results or do otherwise. Finally, this study could be conducted using a different methodology, preferably a quantitative approach to explore the views of a larger group of persons with schizophrenia in the country.

## Data Availability

The raw and analysed data are available with the corresponding author upon reasonable request.
